# Short-Term Percutaneous Mechanical Circulatory Support in Acute Coronary Syndrome with Cardiogenic Shock: Which Device to Choose?

**DOI:** 10.3390/jcdd13020099

**Published:** 2026-02-18

**Authors:** Nardi Tetaj, Annunziata Nusca, Francesco Piccirillo, Geza Halasz, Domenico Gabrielli, Gian Paolo Ussia, Francesco Grigioni

**Affiliations:** 1 Cardiology Unit, Campus Bio-Medico University Hospital, Via Alvaro del Portillo 200, 00128 Rome, Italy; a.nusca@policlinicocampus.it (A.N.); f.piccirillo@policlinicocampus.it (F.P.); g.ussia@policlinicocampus.it (G.P.U.); f.grigioni@policlinicocampus.it (F.G.); 2 Research Unit of Cardiovascular Science, Department of Medicine and Surgery, Università Campus Bio-Medico di Roma, Via Alvaro del Portillo 21, 00128 Rome, Italy; 3 Division of Cardiology, Cardio-Thoracic and Vascular Department, San Camillo Forlanini Hospital, 00152 Rome, Italy; geza.halasz@gmail.com (G.H.); dgabrielli@scamilloforlanini.rm.it (D.G.)

**Keywords:** cardiogenic shock, acute myocardial infarction, mechanical circulatory support, Impella, intra-aortic balloon pump, IABP, extracorporeal membrane oxygenation, VA-ECMO, ECPELLA

## Abstract

Cardiogenic shock (CS) remains a life-threatening syndrome characterized by reduced cardiac output and end-organ hypoperfusion, most commonly resulting from acute myocardial infarction (AMI). Despite advances in early revascularization and increasing use of percutaneous mechanical circulatory support (MCS), short-term mortality in AMI-related CS (AMI-CS) remains high. This review summarizes the contemporary evidence on short-term percutaneous MCS in AMI-CS, with a focus on intra-aortic balloon pump (IABP), Impella microaxial flow pumps, and venoarterial extracorporeal membrane oxygenation (VA-ECMO), and provides insights into device selection and implementation in clinical practice. We performed a comprehensive analysis of the most relevant randomized controlled trials and key guideline recommendations from European and North American societies concerning the use of MCS. Despite its long-standing, IABP has not demonstrated a mortality benefit in contemporary trials and is no longer recommended for routine use in AMI-CS without mechanical complications. Nevertheless, it remains widely used due to its simplicity, safety profile, and broad availability. In contrast, Impella devices provide active left ventricular unloading and have shown promising hemodynamic effects, with the DanGer Shock trial suggesting a potential survival benefit in carefully selected patients, at the expense of higher complication rates. VA-ECMO offers full cardiopulmonary support but is associated with the highest complication rates and increases left ventricular afterload, often requiring adjunctive unloading with devices such as Impella (ECPELLA). However, recent randomized trials have not demonstrated a clear survival advantage for VA-ECMO, and concerns regarding its complications persist. In conclusion, CS continues to pose major therapeutic challenges, and no single MCS device has consistently shown a survival benefit across all AMI-CS patient populations. Individualized, phenotype-driven strategies that incorporate hemodynamic profiling and timely escalation of support are essential. Further randomized studies are urgently needed to define optimal device selection, the timing of placement, and appropriate patient selection criteria. Institutional protocols guided by clinical stage, etiology, and available expertise will be pivotal in improving outcomes.

## 1. Introduction

Cardiogenic shock (CS) is a serious condition characterized by reduced cardiac output and systemic hypoperfusion resulting from primary cardiac dysfunction. According to the Shock Academic Research Consortium (SHARC), CS is defined by a systolic blood pressure of less than 90 mmHg for at least 30 min, or the requirement for vasopressors, inotropes, or mechanical circulatory support (MCS) to maintain a systolic pressure of at least 90 mmHg, accompanied by clinical and biochemical evidence of tissue hypoperfusion [1].

The etiologies of CS include acute myocardial infarction (AMI), fulminant myocarditis, cardiotoxic drug intoxication, end-stage valvular diseases, dilated or ischemic cardiomyopathy, hypothermia with refractory circulatory instability, and massive pulmonary embolism. Among these, AMI is the predominant cause, accounting for approximately 60–80% of cases [2]. CS complicates 6–8% of AMI cases and remains associated with high short-term mortality of up to 50%, despite advancements in MCS technologies and widespread adoption of early revascularization strategies [3,4].

In this context, prompt coronary angiography and revascularization of the culprit lesion remain the cornerstone of treatment and are strongly recommended by both European Society of Cardiology (ESC) and American Heart Association (AHA) guidelines (Class I). However, a substantial subset of patients continues to deteriorate despite optimal revascularization, underscoring the need for adjunctive therapies that can restore systemic perfusion and support myocardial recovery [5]. This has led to an increased use of percutaneous short-term MCS devices, including the intra-aortic balloon pump (IABP), Impella micro-axial flow pumps, and venoarterial extracorporeal membrane oxygenation (VA-ECMO) [6].

According to the 2023 ESC guidelines, short-term MCS may be considered for patients with acute coronary syndrome (ACS), including both STEMI and NSTEMI, complicated by severe or refractory CS (Class IIb, Level C). North American guidelines provide more detailed recommendations. For patients with STEMI who experienced severe or refractory CS, Impella is assigned a Class IIa recommendation with Level of Evidence B-R. In patients with ACS-related mechanical complications and hemodynamic instability, short-term MCS devices are recommended in the North American guidelines (Class IIa, Level B-NR), whereas the ESC guidelines recommend IABP alone (Class IIa, Level C) [7,8]. Importantly, both guidelines discourage the routine use of IABP, and, in the North American guidelines, VA-ECMO for ACS-related CS without mechanical complications, as there is no demonstrated survival benefit (Class III recommendation). These recommendations are summarized in Table 1.

Despite the increasing availability of MCS technologies, clinical decision-making in CS remains highly variable. Large randomized controlled trials have yielded neutral or inconclusive results, fueling ongoing controversy regarding optimal device selection [9]. Furthermore, ESC guidelines do not specify a preferred device, leaving clinicians with limited guidance. Conversely, North American guidelines provide more device-specific recommendations, particularly favoring Impella in STEMI-related CS. This disparity, combined with conflicting trial data, has led to significant variability in clinical practice, often driven by local protocols and institutional experience. Consequently, there is a pressing need for clear, evidence-based algorithms to guide device selection.

In this review, we critically appraise the current evidence surrounding short-term percutaneous MCS in AMI-CS, focusing on randomized controlled trials and contemporary guidelines recommendations. Our goal is to provide clinicians with a comprehensive and practical synthesis to inform the evidence-based, patient-tailored use of MCS in this high-risk population.

## 2. Search Strategy and Selection Criteria

A comprehensive literature search was performed on PubMed and Cochrane covering the period from 2000 to September 2025. The search strategy used the terms acute myocardial infarction, cardiogenic shock, mechanical circulatory support, in combination with key terms related to intra-aortic balloon pump, IABP, Impella, microaxial flow pumps, extracorporeal membrane oxygenation, ECMO, left ventricular unloading, ECPELLA, ECMELLA, TandemHeart, Protek Duo. We excluded proceeding papers, corrections, early access articles, news items, book chapters, retractions, reprints, biographical items, book reviews, meeting abstracts, editorial materials, and letters. We included relevant English-language studies involving adult populations, with particular focus on randomized controlled trials and large observational studies. The full texts were then screened and selected, and the study characteristics and information were extracted from the selected studies.

## 3. Cardiogenic Shock

During the development of a consensus definition of cardiogenic shock (CS), the SHARC Writing Committee proposed two complementary definitions. The first, intended for clinical practice, characterizes CS as a cardiac disorder associated with clinical and biochemical evidence of sustained tissue hypoperfusion. The second, intended for clinical trials, defines CS as a cardiac disorder resulting in a systolic blood pressure of less than 90 mm Hg for at least 30 min, or necessitating vasopressor, inotropic, or mechanical circulatory support to maintain systolic blood pressure of at least 90 mmHg, accompanied by evidence of hypoperfusion [1].

In 2019, the Society for Cardiovascular Angiography and Interventions (SCAI) introduced a staging system to standardize the assessment of CS severity across clinical and research settings. The SCAI classification integrates clinical, biochemical, and hemodynamic parameters into a five-stage model that reflects the dynamic and progressive nature of cardiogenic shock, capturing the continuum of deterioration as in a downward spiral, ranging from simple A (at risk) to complex E (extremis) [9].

**Figure 1:**
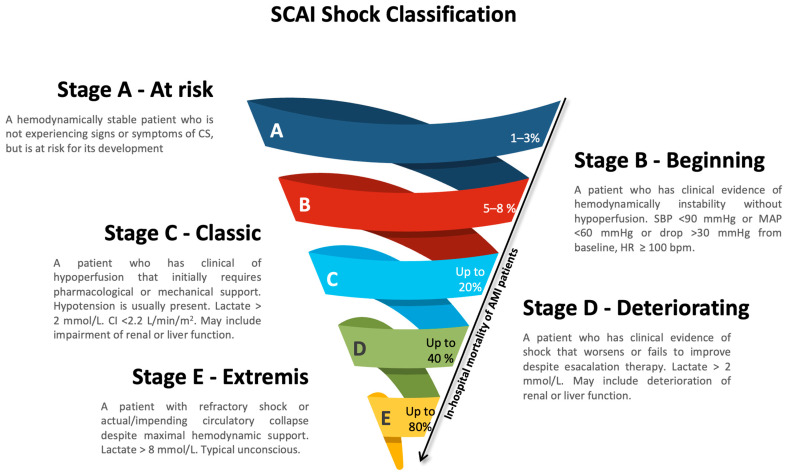
SCAI Shock Classification alongside stage-specific mortality estimates based on current evidence [3,6,10,11,13]. Abbreviations: SCAI, Society for Cardiovascular Angiography and Interventions; CS, cardiogenic shock; SBP, systolic blood pressure; MAP, mean arterial pressure; HR, heart rate; C.I., cardiac index.

Stage A (“at risk”) includes hemodynamically stable patients at risk of developing CS due to acute cardiovascular events such as a large myocardial infarction or early ventricular dysfunction. Stage B (“beginning”) includes patients with early signs of hemodynamic instability but without overt hypoperfusion. Stages C and E are defined by escalating severity of hypoperfusion, evidenced by clinical markers such as cool extremities, oliguria, altered mental status, elevated lactate (>2 mmol/L), and progressive hypotension requiring escalating pharmacologic or mechanical support. Stage E represents profound circulatory collapse, characterized by multiorgan failure and severe derangement (lactate > 8 mmol/L), despite receiving maximal support, including MCS [9] (Figure 1).

**Figure 2:**
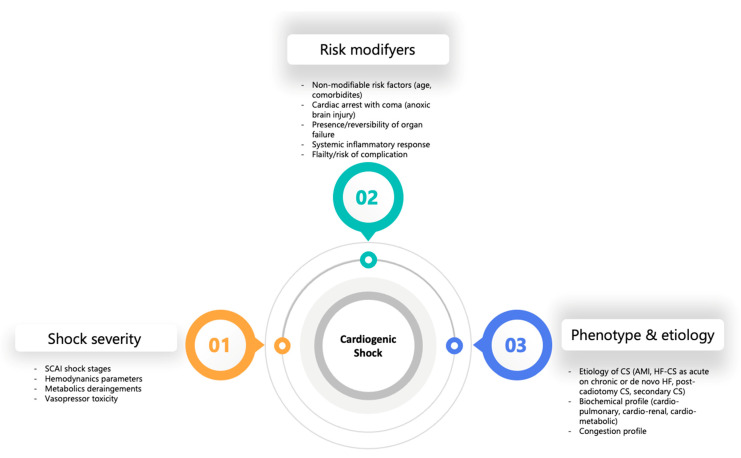
Updated three-axis model of Cardiogenic Shock in the 2022 SCAI Expert Consensus supporting individualized patient management, incorporating predictors of mortality [14]. Abbreviations: SCAI, Society for Cardiovascular Angiography and Interventions; CS, cardiogenic shock; AMI, acute myocardial infarction; HF, heart failure.

In 2022, an expert consensus refined the SCAI classification, incorporating clinical insights and validating its prognostic utility. Subsequent validations have consistently demonstrated a stepwise increase in mortality with advancing SCAI shock stages, although the specific mortality rate associated with each stage has varied across studies and is not strictly linear [10,11,12]. These refinements introduced a three-axis model to facilitate individualized risk stratification and incorporating predictors of mortality: (1) shock severity, represented by SCAI stage; (2) risk modifiers, including advanced age, comorbidities, anoxic brain injury, systemic inflammatory response, or frailty; and (3) shock phenotypes and etiology, encompassing clinical profile such as congestion, biochemical phenotype (e.g., cardio-renal or cardiometabolic) and underlying causes such as AMI-CS, HF-CS (acute on chronic or de novo HF), post-cardiotomy CS, and secondary causes (e.g., arrhythmia, valvular or pericardial disease) [13,14]. This framework aids in both clinical decision-making and research design, aligning interventions with patient-specific profiles (Figure 2).

## 4. Intra-Aortic Balloon Pump

Introduced by Kantrowitz in 1967, the intra-aortic balloon pump (IABP) was the first mechanical circulatory support (MCS) device adopted into routine clinical use. Its simplicity, safety profile, and cost-effectiveness made it the most widely utilized MCS device for decades [15]. With the expansion of percutaneous coronary intervention (PCI) in the 1980s, IABP use increased, particularly for cardiogenic shock and high-risk PCI [16].

The IABP consists of a 7–8 Fr double-lumen catheter with a 20–50 mL balloon positioned in the descending thoracic aorta, below the left subclavian artery. The balloon is timed to inflate during diastole (typically during the T-P interval) and deflate during systole (QRS-T interval), enhancing coronary perfusion and reducing afterload. Helium is used for rapid inflation and deflation due to its low density and inert properties. The system is synchronized at a 1:1 ratio with the cardiac cycle but may be adjusted (e.g., 2:1) during weaning [17,18].

Hemodynamically, IABP augments diastolic pressure, improves coronary perfusion, and reduces left ventricular (LV) end diastolic pressure, thereby decreasing afterload and increasing stroke volume and cardiac output [18]. Despite these physiologic benefits, randomized trials have not translated them into a proven survival benefit in either AMI-CS or HF-CS [19].

The IABP-SHOCK I trial (2010) was a single-center randomized clinical trial (RCT) conducted in Germany, enrolling 45 patients with AMI complicated by CS undergoing PCI. Recruitment took place between March 2003 and June 2004. Patients were randomized to receive either standard care with IABP or without IABP. The study found non-significant improvements in cardiac index and inflammatory markers four days after enrollment; however, these findings were limited by the small sample size and lack of statistical power [20]. Similarly, the randomized, multicenter CRISP AMI trial (2011) evaluated the role of IABP in 337 patients with acute anterior STEMI without CS. No significant reduction in infarct size was observed in this trial [21].

The IABP-SHOCK II (2012) was a pivotal German multicenter RCT, comparing IABP vs. standard care in AMI-CS patients undergoing primary PCI. Between June 2009 and March 2012, 600 patients were randomized to either routine IABP support or standard care. No significant differences were observed in 30-day, 1-year, and 6-year mortality, or in secondary outcomes (reinfarction, stroke, bleeding, renal function). The trial noted that IABP was often placed post-PCI and included patients with relatively mild shock (25% had lactate levels < 2 mmol/L), which may have limited its effectiveness. Additionally, a high rate of pre-randomization cardiac arrest (45%) may have confounded mortality outcomes due to anoxic brain injury [22,23].

**Figure 3:**
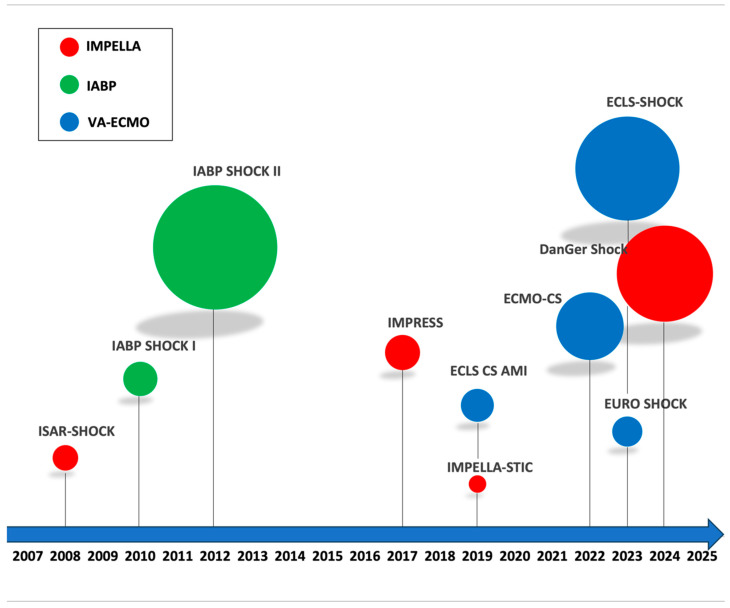
The Timeline of randomized controlled trials evaluating mechanical circulatory support (MCS) in patients with acute myocardial infarction (AMI) complicated by cardiogenic shock (CS) undergoing percutaneous coronary intervention (PCI). The size of each circle is proportional to the number of patients enrolled. Red circles indicate trials in which Impella was used as the MCS intervention, green circles denote trials using IABP, and blue circles represent trials in which VA-ECMO was the intervention. Abbreviations: AMI, acute myocardial infarction; CS, cardiogenic shock; PCI = percutaneous coronary intervention; MCS, mechanical circulatory support; IABP = intra-aortic balloon pump; VA-ECMO = venoarterial extracorporeal membrane oxygenation.

These clinical trials, along with other RCTs evaluating MCS devices in AMI-related CS patients undergoing PCI, are summarized and visually depicted in the timeline bubble chart shown in Figure 3.

Until 2012, both ESC and ACC/AHA guidelines recommended IABP as a Class I therapy for AMI-CS. However, following the IABP-SHOCK II trial, this recommendation was downgraded to Class III (not recommended) for routine use in AMI-CS without mechanical complications, due to a lack of benefit. IABP remains recommended (Class IIa, Level C) in AMI-CS with mechanical complications (e.g., free wall rupture, papillary muscle rupture), based on expert consensus [7,8]. Despite the lack of demonstrated mortality benefit, IABP continues to be widely used due to its ease of use, safety, and availability.

## 5. Microaxial Flow Pump

The Impella device is a percutaneous, catheter-based microaxial ventricular assist device designed to pump blood from the left ventricle (LV) into the ascending aorta. Depending on the model, it can provide flow rates ranging from 2.5 to 5.0 L/min. The device operates on the principle of Archimedes’ screw, historically used in Ancient Egypt to lift water from the Nile River for irrigation purposes. Its motor generates rotational force, creating a negative pressure that draws blood into the inflow area and propels it forward through the cannula to the outflow tract in the ascending aorta, actively unloading the LV. The Hemopump, a precursor of the current device, was developed in the 1980s by Dr. Richard Wampler, who was inspired by irrigation pumps he saw during a medical mission in Egypt. In the 1990s, Siess and colleagues in Aachen, Germany, refined the design to create a more efficient percutaneous device, and the Impella system received European approval in 2005. In the U.S., the FDA approved the Impella 2.5 in 2008 and subsequently expanded its approval in 2016 to the full Impella family for left-sided heart use, including 2.5, CP (cardiac power), 5.0, and 5.0/LD for use in cardiogenic shock [24,25]. The system is operated through the Automated Impella Controller (AIC), which provides real-time adjustments of the performance level (P-level) based on patient needs [26].

Hemodynamically, the device reduces LV stroke work, myocardial oxygen demand, and pulmonary artery wedge pressure (PAWP), while improving coronary blood flow, mean arterial pressure, and peripheral organ perfusion [27].

Early randomized trials, such as ISAR-Shock (2008), which included 26 patients with AMI-CS undergoing primary PCI, showed superior hemodynamic improvements with Impella LP2.5 over IABP after 30 min of mechanical support. However, no mortality benefit was observed [28]. The IMPRESS trial (2017), a two-center randomized study conducted in Amsterdam and Bergen between June 2012 and September 2015, enrolled 48 mechanically ventilated STEMI-CS patients undergoing primary PCI. The study found no significant difference in all-cause mortality at 30 days or 6 months between Impella CP and IABP. Notably, 92% of patients experienced cardiac arrest prior to randomization, suggesting a high rate of post-anoxic brain injury at the time of randomization, potentially limiting the clinical benefit of mechanical support in these patients [29]. While the 5-year follow-up revealed no difference in all-cause mortality, the major adverse cardiac and cerebrovascular events (MACCE) rate was higher in the IABP group [30]. The Impella STIC trial (2019), a French randomized study involving 12 patients, found no hemodynamic benefit when escalating support from IABP to Impella LP5.0 in stabilized patients and observed an increased risk of bleeding [31]. Collectively, these early trials were limited by their small sample sizes and insufficient statistical power to detect significant differences in clinical outcomes.

The DanGer Shock trial, involving 355 STEMI-CS patients across centers in Denmark, Germany, and the United Kingdom, was the first RCT to demonstrate a mortality benefit with Impella CP. At 180 days, mortality was significantly lower in the Impella group than in the standard care group (45.8% vs. 58.5%, *p* = 0.04). However, this benefit came at the cost of increased complications such as bleeding, limb ischemia, and the need for renal replacement. Although the trial achieved its target sample size and reached 80% statistical power, it had some limitations. The strict inclusion and exclusion criteria limit the generalizability of its findings. Furthermore, the study was conducted over 10 years (January 2013 to July 2023), raising the possibility of temporal changes in clinical practice that may have influenced the outcomes [32].

A long-term follow-up of the Danger Shock Trial showed that the survival benefit appeared sustained for up to 10 years after randomization [33].

A 2019 European multinational retrospective matched-pair analysis compared outcomes in 237 patients with AMI-CS treated with Impella with those of 237 matched patients from the IABP-SHOCK II trial cohort. The study found no significant difference in 30-day all-cause mortality between the two groups. However, it reported higher complication rates with the Impella device, including bleeding and peripheral vascular complications [34].

The Impella ECP (Expandable Cardiac Power) is a novel 21 Fr device that can be compressed to 9 Fr for easier insertion and removal. In 2020, U.S. physicians treated the first patient with this device. Capable of delivering up to 5.5 L/min, it is currently under investigation in two prospective trials: ECP EFS (NCT04477603) and ECP Study (NCT05334784), which assess safety and MACCE outcomes in high-risk PCI [35].

The Impella RP is a percutaneous right ventricular (RV) assist device designed to deliver blood from the right atrium to the pulmonary artery. Despite its innovative approach, clinical evidence supporting its use remains limited. Initial data from the RECOVER RIGHT trial (2015) showed that the Impella RP was safe, easy to deploy, and reliably resulted in immediate hemodynamic benefit in patients with life-threatening RV failure. This trial reported an overall 30-day survival rate of 73.3% [36]. However, the subsequent 2019 FDA post-approval surveillance revealed higher-than-expected mortality, prompting a safety advisory [37].

## 6. Venoarterial Extracorporeal Membrane Oxygenation

Extracorporeal membrane oxygenation (ECMO) is a form of temporary mechanical cardiorespiratory support that has evolved considerably since its first successful introduction in the 1970s by Robert Bartlett. Reflecting its increasing adoption, the Extracorporeal Life Support Organization (ELSO), the world’s largest ECMO patient registry and network, published its first official guidelines for ECMO use in 2005 [38]. Veno-venous ECMO (VV-ECMO) is specifically designed to provide full respiratory support in cases of severe respiratory failure, such as acute respiratory distress syndrome (ARDS) [39]. In contrast, venoarterial ECMO (VA-ECMO) offers full systemic support, providing both circulatory and respiratory assistance. This is achieved by draining blood via a large-bore venous cannula, passing it through a centrifugal device, oxygenating it, and returning it to an arterial cannula. International guidelines support VA-ECMO use in carefully selected patients with CS as a bridge to recovery or transplantation. These recommendations are primarily based on current evidence and expert opinion [40,41].

Current evidence specifically addressing percutaneous VA-ECMO in AMI-CS has strengthened in recent years, supported by four RCTs—ECLS SHOCK I [42], EURO SHOCK [43], ECMO-CS [44], and ECLS-SHOCK [45]. These studies used modern ECMO devices, reflecting contemporary technology and practice.

The ECLS SHOCK I (or ECLS CS AMI) trial (2019) was a single-center randomized study in Munich that investigated the use of VA-ECMO in 42 AMI-CS patients. The trial found no significant difference in 30-day LV ejection fraction or secondary outcomes (such as stroke, bleeding, peripheral ischemic complications, or reinfarction) between VA-ECMO and standard therapy [42].

The ECMO-CS trial (2023), a Czech multicenter study, enrolled 122 patients with rapidly deteriorating severe CS (stages D/E) from various etiologies, with 65% of cases related to AMI. Patients were treated either with immediate VA-ECMO or with no immediate VA-ECMO. No difference in the 30-day composite of all-cause mortality, resuscitated circulatory arrest, or the implementation of another MCS device was observed between the two treatment groups. Similarly, no differences were observed in the individual components of the primary endpoint, including all-cause mortality and resuscitated cardiac arrest. Safety outcomes, such as adverse event incidence, also did not differ significantly between groups. However, 39% of patients in the early conservative group ultimately required downstream VA-ECMO support, potentially diluting the differences between the groups. Furthermore, the trial was powered only to detect differences in the composite primary outcome, limiting its ability to identify statistically significant differences in individual components, secondary outcomes, or subgroup analyses. An additional limitation was the presumed 50% reduction in the primary endpoint, which may have been excessive and led to an overestimation of the anticipated benefit and to underpowering for more modest but clinically meaningful differences [44].

The EURO SHOCK (2023) study was a multicenter European trial that enrolled 35 patients with AMI and CS. Participants were randomized to receive either VA-ECMO or standard therapy. The study found no significant differences in all-cause mortality between the two groups at 30 days or 12 months. However, VA-ECMO was associated with more vascular and bleeding complications. Notably, the trial faced several limitations. Recruitment was significantly hampered by the COVID-19 pandemic, with less than 10% of the originally planned sample size enrolled. In addition, a high crossover rate between groups may have further confounded treatment outcomes. As a result, no definitive conclusions could be drawn from the study [46].

Finally, in 2023, the largest RCT to date, the ECLS-SHOCK trial (2023), was published. It included 420 patients with AMI-CS undergoing revascularization (95.7% PCI, the other CABG). Participants were randomized to receive either early VA-ECMO vs. standard medical therapy alone, with escalation to other forms of MCS—such as IABP or Impella—if deemed necessary. No significant difference in 30-day or 1-year all-cause mortality was found (47.8% in the ECLS group vs. 49% in the control group). Nevertheless, similar to previous studies, VA-ECMO was associated with higher bleeding and vascular complications. Despite being a well-executed trial that also used post hoc classification of CS stages and being the only study powered to detect a difference in mortality, several limitations should be acknowledged. Importantly, a high proportion of resuscitated patients before randomization (78%) was included, and a quarter of patients were older than 69 years. This does not align with current global practice, as many centers consider age above 65 or 70 years a relative contraindication for VA-ECMO implantation. In addition, the high crossover rate of patients to the VA-ECMO group may have diluted the treatment effect, potentially underestimating the benefit of early VA-ECMO initiation [45,47]. Interestingly, a recent subanalysis of the ECLS-Shock trial demonstrated that all-cause 30-day mortality was higher in patients receiving bailout and escalated MCS therapy than in those receiving upfront VA-ECMO only [48].

Further support for the lack of routine benefit of VA-ECMO in AMI-CS comes from an individual patient data (IPD) meta-analysis that pooled results from all four RCTs, including 567 patients with AMI-CS. The analysis confirmed the absence of a 30-day mortality benefit among patients receiving routine VA-ECMO and highlighted an increased risk of bleeding and vascular complications with VA-ECMO [43].

**Figure 4:**
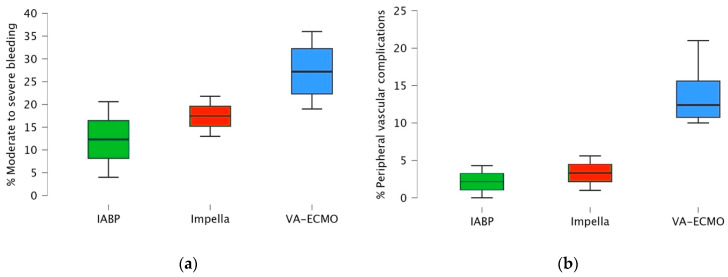
Box plot illustrating the complication rates of: (**a**) moderate to severe bleeding; (**b**) peripheral vascular complications; associated with each mechanical circulatory support device, as reported in major randomized controlled trials to date, IABP-SHOCK II [22], IMPRESS [30], DanGer Shock [32], EURO SHOCK [43], ECMO-CS [44], and ECLS-SH.OCK [45].

A comparison of the contraindications and complications associated with available short-term percutaneous MCS devices—namely, IABP, Impella, and VA-ECMO—is summarized in Table 2. A stepwise increase in complication rates, particularly bleeding and vascular events, is evident from IABP to Impella and subsequently, to VA-ECMO, as reported in major RCTs to date (IABP-SHOCK II, IMPRESS, DanGer Shock, ECLS-SHOCK, ECMO-CS, and EURO SHOCK). This trend, illustrated in the box plots in Figure 4, is primarily attributable to differences in vascular access cannula size and the insertion technique required for each device.

## 7. Left Ventricular Unloading Strategies During VA-ECMO

Peripheral VA-ECMO returns oxygenated blood into the femoral artery, which flows retrograde (upward) into the descending and ascending aorta. This retrograde perfusion increases aortic pressure, thereby increasing LV afterload. In patients with severely impaired LV function, as in cardiogenic shock, LV may be unable to generate sufficient pressure to overcome this increased afterload and open the aortic valve. As a result, the aortic valve may remain closed, blood slows down, and accumulates within the LV, leading to progressive LV distension. This distension increases left atrial and pulmonary venous pressures, contributing to pulmonary congestion, elevated pulmonary capillary wedge pressure, pulmonary edema, and impaired gas exchange despite adequate systemic oxygenation. Furthermore, blood stasis within the LV cavity and aortic root promotes thrombus formation, which poses a significant risk for systemic embolization, including stroke. To mitigate these complications, LV unloading strategies are often employed, including IABP, Impella, or atrial septostomy [49,50].

Among these available strategies, the Impella device is most commonly used, enabling simultaneous LV venting during VA-ECMO support, a configuration referred to as ECPELLA. However, observational data suggest a potential survival benefit with LV unloading, despite a higher incidence of major bleeding events [51,52,53]. To date, no randomized controlled trial has definitively validated the clinical efficacy of LV unloading.

The IABP and Impella differ significantly in their mechanisms of action and, consequently, in their effects on LV pressure and volume. The IABP increases aortic diastolic pressure during diastole, enhancing coronary perfusion, and indirectly reduces LV pressure by lowering aortic systolic pressure and afterload during systole. In contrast, the Impella actively aspirates blood from the LV and expels it into the ascending aorta, providing direct LV unloading by decreasing both LV volume and pressure. Compared to the IABP, the Impella has a more pronounced effect on reducing LV pressure and volume, although IABP’s impact on afterload reduction is relatively modest [54,55].

The EARLY-UNLOAD trial (2023) enrolled 116 patients with CS—66% of whom had AMI—who received VA-ECMO support, at Chonnam National University Hospital, South Korea. Patients were randomized to either early routine LV unloading via transseptal left atrial cannulation within 12 h of randomization or to a conventional management strategy. The primary endpoint, 30-day all-cause mortality, did not differ significantly between the two groups [51].

The HERACLES trial (Hemodynamic Effects of Reducing Left Ventricular Afterload With Impella or IABP During VA-ECMO in CS; ISRCTN82431978) is an ongoing, single-center study expected to conclude in November 2026. This trial will randomize 36 CS patients receiving VA-ECMO and LV unloading with either an IABP or Impella, aiming to compare the physiological effects of each device on coronary perfusion and ventricular pressures and volumes. In contrast, the REVERSE trial, a multicenter RCT evaluating 45-day MCS-free survival, was terminated prematurely in 2023 due to poor enrollment.

An ongoing RCT (UNLOAD-ECMO; NCT05577195) compares VA-ECMO combined with Impella versus VA-ECMO alone in patients with CS of all causes. The results of this trial will be pivotal in determining whether combined ECMO and Impella therapy is clinically justified. Another ongoing RCT, ANCHOR (NCT04184635), aims to evaluate the superiority of VA-ECMO combined with IABP compared to optimal medical therapy alone in AMI-CS patients.

## 8. Other Mechanical Cardiac Support Devices

The TandemHeart system is a percutaneous, centrifugal ventricular assist device designed to unload the failing left ventricle by diverting oxygenated blood from the left atrium to the distal descending aorta. It consists of four main components: a control console, a 21F transseptal inflow cannula placed in the left atrium, a 15–19F arterial outflow cannula, and a centrifugal pump driven by an electromagnetic motor, which can deliver up to 5.0 L/min. The TandemHeart device, in conjunction with the left ventricle, enhances forward blood flow, thereby reducing LV pressure, volume, and stroke volume. However, clinical data on TandemHeart remain limited. Two early randomized trials, conducted between 2005 and 2006, including patients with CS, demonstrated superior hemodynamic parameters, such as higher cardiac power index and lower pulmonary capillary wedge pressure, compared to intra-aortic balloon pump (IABP) [56,57]. However, these benefits were offset by higher rates of severe complications, including major bleeding and limb ischemia. An alternative to the TandemHeart for right ventricular (RV) support is the PROTEK Duo dual-lumen cannula. This 29F cannula is inserted via the right internal jugular vein and provides percutaneous RV support by enabling simultaneous venous drainage from the right atrium and reinfusion into the pulmonary artery. Although the PROTEK Duo offers a minimally invasive option for RV failure, evidence regarding its safety and efficacy remains limited, and further prospective studies are needed to clarify its clinical utility [58].

**Figure 5:**
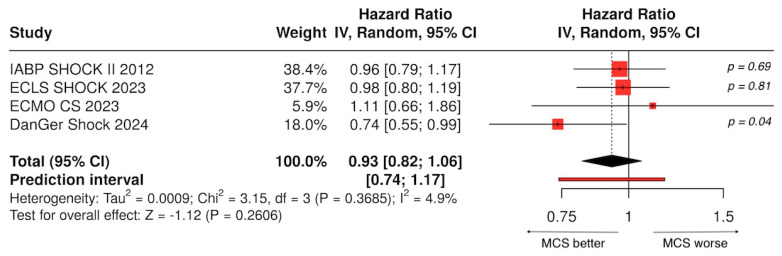
Hazard ratios for 30-day all-cause mortality were reported in the IABP-SHOCK II [22], ECLS-SHOCK [45], and ECMO-CS trials [44], while 180-day mortality was assessed in the DanGer Shock trial [32]. All studies achieved the anticipated sample sizes, reaching 80% statistical power to provide robust estimates of treatment effects.

Table 3 summarizes the main RCTs investigating MCS devices in AMI-related CS, including at least 25 patients undergoing primary PCI, and is visually illustrated in the forest plot in Figure 5.

## 9. Practical Algorithm for Device Selection and Timing

The management of AMI-related cardiogenic shock (AMI-CS) requires timely identification of the underlying pathophysiology, rapid hemodynamic stabilization, and individualized device selection. Despite significant advances, the absence of a universally validated algorithm often results in heterogeneous practice patterns across institutions. A structured, stage-oriented approach integrating SCAI classification, hemodynamic profiling, and institutional resources is essential to optimize outcomes.

In SCAI stage B, patients with hypotension or early signs of hypoperfusion but preserved organ function generally respond to pharmacologic therapy, and routine MCS implantation is not indicated. Stage C is characterized by sustained hypotension, elevated lactate, and early organ dysfunction, and represents the optimal “therapeutic window” for initiating percutaneous MCS, before multiorgan failure develops. In stage D (“deteriorating”), cardiogenic shock fails to improve despite escalation of therapy; thus, MCS should be strongly considered. Stage E (“extremis”) denotes refractory shock despite maximal therapy. In this setting, MCS may serve as rescue therapy or a bridge to decision, although mortality remains exceedingly high [59].

Current evidence supports early or pre-PCI MCS insertion in selected AMI-CS patients, as delayed or bailout implantation is associated with worse outcomes. In a nationwide analysis of 161,789 AMI-CS cases, early (<24 h) initiation of MCS was linked with reduced mortality and shorter hospitalization compared with delayed implantation [60,61]. Similarly, subgroup analyses from the DanGer Shock and ECLS-SHOCK trials suggest that upfront MCS prior to revascularization may preserve end-organ perfusion and limit ischemia–reperfusion injury [48,61].

Therefore, MCS should ideally be deployed prior to PCI, during stage C or early stage D, as this timing appears to offer the greatest potential for myocardial recovery and survival benefit. In contrast, late initiation after systemic collapse is often futile.

Once myocardial recovery or hemodynamic stability is achieved, we recommend stepwise weaning from high-flow to lower-support devices, guided by lactate clearance, cardiac index (>2.2 L/min/m^2^), and mean arterial pressure (>65 mmHg).

The implementation of MCS in AMI-CS should be coordinated by a dedicated shock team comprising interventional cardiologists, intensivists, and perfusionists to ensure prompt decision-making and minimize delays.

In real-world clinical settings, logistical coordination and mobilization of the multidisciplinary team can introduce significant delays, a critical issue in AMI-CS where the therapeutic window for timely revascularization is extremely narrow.

Accordingly, early CS recognition, prompt initiation of tailored temporary MCS, and serial reassessment are key in improving outcomes [62]. Evidence of pulmonary edema, persistent congestion, worsening perfusion, and/or multiorgan dysfunction should prompt a team-based discussion regarding escalation of tMCS such as ECPELLA.

Centers with established MCS programs demonstrate better outcomes through structured algorithms, predefined activation pathways, and greater expertise in patient selection, cannulation, and weaning.

**Figure 6:**
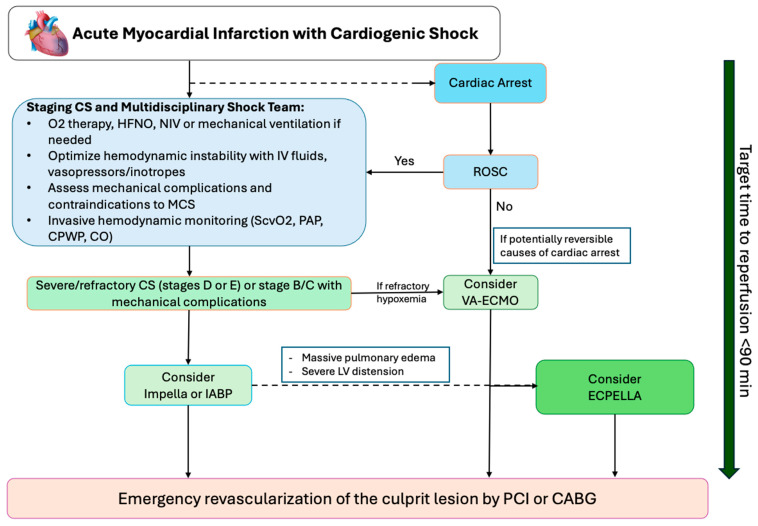
Example of a simplified algorithm for selecting short-term percutaneous MCS in CS-AMI. Abbreviations: O2, oxygen; HFNO, high flow nasal oxygenation; NIV, noninvasive ventilation; IV, intravenous; CS, cardiogenic shock; LV, left ventricle; PCWP, pulmonary capillary wedge pressure; ScvO2, central venous oxygen saturation; CO, cardiac output; IABP, intra-aortic balloon pump; ROSC, return of spontaneous circulation; PCI, percutaneous coronary intervention; CABG, coronary artery bypass grafting.

A simplified algorithm for selecting short-term percutaneous MCS in patients with AMI-related cardiogenic shock is illustrated in Figure 6.

## 10. Discussion

Cardiogenic shock (CS) remains one of the most challenging conditions in contemporary cardiovascular medicine. Despite advances in pharmacological therapies, revascularization techniques, and mechanical circulatory support (MCS) technologies, short-term mortality rates remain unacceptably high, often exceeding 40–50% in AMI-related CS [63]. Early recognition and timely intervention are crucial; however, defining the optimal management strategies is complicated by the lack of high-level evidence and significant heterogeneity in clinical practice [64].

The increasing use of percutaneous MCS devices, such as IABP, Impella, and VA-ECMO, highlights the need for circulatory stabilization in critically ill patients [55]. While each of these devices offers specific hemodynamic advantages, none has consistently demonstrated a survival benefit in unselected CS populations.

To date, only a few RCTs have been completed in MCS patients with AMI-CS undergoing primary PCI, as shown in Table 4. These landmark trials have been pivotal in refining device indications and contraindications, while highlighting the importance of appropriate patient selection and timing of support initiation. The DanGer Shock was the only RCT to provide a potential signal of improved survival with early Impella CP use in AMI-CS, albeit at the cost of higher complication rates. Moreover, the use of ECPELLA, combining VA-ECMO with active LV unloading, has shown promising physiological effects in observational studies, even though it is associated with a high complication rate. This approach is currently being evaluated in ongoing trials [65,66,67]. Meanwhile, the emergence of newer devices, such as the Impella ECP, reflects ongoing innovation in percutaneous support.

Regarding the indications for MCS implantation, international guidelines remain partially discordant. While the ESC guidelines offer a broad framework with limited device-specific recommendations, North American guidelines have taken a more prescriptive approach, especially in favoring Impella for STEMI-related CS, based on the results of the latest DanGer Shock trial. However, both recommend against routine IABP use in AMI-CS without mechanical complications and stress the importance of individualized, multidisciplinary decision-making. A noticeable increase in device complication rates is observable from IABP to Impella, and then to VA-ECMO, primarily attributable to differences in vascular access cannula size, insertion techniques, and pump mechanisms. This trend is supported by findings in the current literature [68,69,70] and is summarized in Table 2 and Figure 4.

As shown in Figure 5, a forest plot of four main RCTs with 80% statistical power, only the DanGer Shock trial demonstrated a statistically significant decrease in 180-day mortality among patients treated with Impella. The DanGer Shock trial highlights an important conceptual issue: its survival benefit likely reflects not only the Impella device, but also the trial’s strict inclusion criteria, early identification of shock, and highly standardized workflow. These elements inherently represent a form of structured triage, prioritizing patients in reversible stages of shock (mostly SCAI C–D) and excluding those with advanced multiorgan failure. Ethically, such triage is justified when used to direct resource-intensive therapies toward patients with the highest probability of benefit. Current consensus statements similarly emphasize that advanced MCS may be inappropriate in profound metabolic collapse or irreversible organ injury, underscoring the need for clear institutional criteria and Shock Team governance [33].

Cost-effectiveness is a key component of MCS decision-making. The IABP-SHOCK II economic analysis demonstrated no cost-effectiveness advantage for routine IABP use, illustrating how increased resource utilization without survival benefit can affect practice [71]. Although comprehensive economic data for Impella and VA-ECMO remain limited, existing analyses suggest substantially higher costs driven by device price and complication management. These considerations further support the need for targeted, stage-appropriate MCS use, ensuring that resource-intensive support is offered to patients most likely to benefit.

Regional differences in regulatory approval, reimbursement, and MCS infrastructure also influence device choice and timing. In the United States, broader access to Impella and organized shock teams favors earlier LV unloading strategies. In contrast, in many European centers, the greater availability of VA-ECMO within cardiac surgery or ICU networks often supports its use as rescue therapy in profound or post–arrest shock. These contextual factors partly explain differences between European and North American guideline recommendations and should be considered when translating evidence into local practice.

Furthermore, the growing complexity of MCS necessitates the implementation of institutional protocols that integrate clinical, hemodynamic, and biochemical markers—such as SCAI staging, comorbidities, and underlying etiologies—to guide timely and appropriate device selection.

A nationwide observational study (2023)—analyzing data from 161,789 AMI-CS patients treated between 2016 and 2020—compared outcomes based on early (<24 h) vs. delayed (≥24 h) initiation of MCS using IABP (26.4%), Impella (11.4%), or VA-ECMO (2.7%). The results indicated that early MCS was associated with fewer complications, shorter hospital stays, and lower rates of 30-day mortality [60]. However, the study also had several limitations. The decision to initiate MCS and the 24-h cut-off was clinically arbitrary, and the analysis was subject to potential confounding bias inherent to observational study designs.

Despite these encouraging results, a structured approach that aligns patient phenotype, shock severity, and device-specific characteristics remains essential for optimizing outcomes [72,73].

Future research should focus on randomized trials adequately powered to evaluate clinically meaningful endpoints. Additionally, it is important to gain a deeper understanding of patient phenotypes that may benefit most from these devices, as well as those who may experience higher complication rates. Finally, identifying biomarkers that can predict patient responses to specific mechanical circulatory support (MCS) strategies is crucial. Moreover, technological advances, such as the development of devices with smaller vascular profiles (e.g., the Impella ECP), may help reduce complication rates and improve the safety and feasibility of MCS deployment.

In conclusion, the management of cardiogenic shock is evolving from an empiric, reactive model to a more structured, evidence-informed, and patient-centered paradigm. Early, stage-specific, and multidisciplinary application of MCS, rather than late or empiric use, appears crucial to improving survival in AMI-related cardiogenic shock. Integration of standardized algorithms into institutional protocols represents the next step toward precision-guided shock management.

As technology advances and our understanding of CS deepens, integrating MCS into personalized care pathways will be critical to reducing morbidity and mortality in this high-risk population.

## Figures and Tables

**Table 1:** Summary of current guideline recommendations from major European and North American cardiology societies regarding mechanical circulatory support (MCS) in patients with acute coronary syndrome (ACS) complicated by cardiogenic shock (CS).

Cardiology Societies	Recommendation
**ACC, AHA, SCAI (2025)**	In patients with mechanical complications of ACS, short-term MCS devices are reasonable for hemodynamic stabilization as a bridge to surgery (CoR 2a, LOE B-NR)
**ACC, AHA, SCAI (2025)**	In selected patients with STEMI and severe or refractory CS, insertion of a microaxial intravascular flow pump is reasonable to reduce death (CoR 2a, LOE BR).
**ESC (2023)**	In patients with ACS and severe/refractory CS, short-term mechanical circulatory support may be considered (CoR IIb, LOE C).
**ESC (2023)**	IABP should be considered in patients with hemodynamic instability/cardiogenic shock due to ACS-related mechanical complications (CoR IIa, LOE C).
**ESC (2023)**	The routine use of an IABP in ACS patients with CS and without mechanical complications is not recommended (CoR III, LOR B)
**ACC, AHA, SCAI (2025)**	In patients with AMI and cardiogenic shock, the routine use of IABP or VA-ECMO is not recommended due to a lack of survival benefit (CoR 3, LOE B-R)

Abbreviations: ESC, European Society of Cardiology; ACC, American College of Cardiology; AHA, American Heart Association; SCAI, Society for Cardiovascular Angiography and Interventions; IABP, intra-aortic balloon pump; VA-ECMO, venoarterial extracorporeal membrane oxygenation; AMI, acute myocardial infarction; STEMI, ST-elevation myocardial infarction; CoR, class of recommendation; LOE, level of evidence.

**Table 2:** Comparison of available devices for the main short-term percutaneous mechanical circulatory support.

	IABP	Impella	VA-ECMO
Pump mechanism	Balloon inflation-deflation	Axial-flow continuous pump	Centrifugal-flow continuous pump
Cannula placement	Descending thoracic aorta	Left ventricle-to-Ascending Aorta (CP/5.5); Right Atrium-to-Pulmonary Artery (RP)	Right Atrium-to-Aorta
Peripheral resistance	Decreased	Mildly increased	Increased
Cardiac output	0.5–1 L/min	2.5–5.5 L/min	4–7 L/min
Arterial femoral access	7–8 Fr	13 Fr (2.5), 14 Fr (CP), 21 Fr (5.0 and 5.5), 9 Fr (ECP)	14–22 Fr
Venous access	None	None	17–28 Fr
Contraindications			
	Severe aortic regurgitation	Severe aortic regurgitation	Severe aortic regurgitation
	Aortic dissection	Aortic dissection	Aortic dissection
	Severe peripheral arterial disease	Severe peripheral arterial disease	Severe peripheral arterial disease
		Absolute contraindications to anticoagulation	Absolute contraindications to anticoagulation
		LV thrombus	
		Mechanical aortic valve	
		Severe aortic stenosis	
General complications			
Moderate/severe bleeding	+	++	+++
Vascular complications	+	++	+++
Thrombocytopenia	+	+	++
Hemolysis	+	++++	++
Specific complications			
	Spinal cord ischemia	Ventricular arrhythmias	Harlequins syndrome
	Device dislocation	Myocardial perforation	Pulmonary edema
		Device dislocation	LV dilatation
			LV stasis and thrombus
			Systemic gas embolism
			Clot formation in the circuit

Abbreviations: Ao, aorta; IABP: intra-aortic balloon pump; LV: left ventricle/ventricular; RV: right ventricle; VA-ECMO: venoarterial extracorporeal membrane oxygenation; +, ++, +++, and ++++ indicate increasing severity of the corresponding complications.

**Table 3:** Key randomized controlled trials of mechanical circulatory support in AMI-related CS undergoing PCI.

Trial (Year)	Sample Size	Intervention vs. Control	Population	Inclusion CS Criteria	Primary Outcome	30-Day All-Cause Mortality MCS Group	Moderate-to-Severe Bleeding	Peripheral Vascular Complications
IABP SHOCK I (2010) [20]	45	IABP vs. OMT	AMI-CS	Hemodynamic instability and end-organ hypo-perfusion, CI < 2.2 L/min/m^2^.	No difference in APACHE II score and CI at 4 days	36.8%	NR	NR
IABP SHOCK II, (2012) [22]	600	IABP vs. OMT	AMI-CS	Hemodynamic instability and end-organ hypo-perfusion, symptoms and signs of pulmonary congestion, lactate level > 2.0 mmol/L.	No difference in 30-day all-cause mortality	39.7%	20.6%	4.3%
ISAR-SHOCK (2008) [28]	25	Impella vs. IABP	AMI-CS	Hemodynamic instability and end-organ hypo-perfusion, CI < 2.2 L/min/m^2^.	Improvement in Cardiac Index after 30 min of support with Impella LP2.5	46% in both groups.	NR	NR
IMPRESS (2017) [29]	48	Impella CP vs. IABP	STEMI-CS	Mechanically ventilated patients with severe CS defined as hemodynamic instability.	No difference in 30-day all-cause mortality	Impella: 46% IABP: 50%	Impella: 13% IABP: 4%	Impella: 1% IABP: 0%
DanGer Shock, (2024) [32]	360	Impella CP vs. OMT	STEMI-CS	Best corresponds to SCAI stages C, D, or E: Hemodynamic instability and end-organ hypoperfusion, with an arterial lactate level > 2.5 mmol/L, and a LV ejection fraction < 45%.	Significantly lower risk of all-cause mortality at 180 days, in the Impella group	39.6%	21.8%	5.6%
ECLS CS AMI, (2019) [42]	42	VA-ECMO vs. OMT	AMI-CS	Hemodynamic instability and end-organ hypoperfusion, with an arterial lactate level > 3 mmol/L.	No difference in LV EF at 30 days	33%	19%	10%
ECMO-CS (2023) [44]	122	VA-ECMO vs. OMT	CS of various causes (65% of which were AMI-related)	Best corresponds to SCAI stages D/E: Rapidly deteriorating shock or severe shock, defined as progressive hemodynamic instability needing vasopressors to maintain SBP > 50 mmHg, arterial lactate > 3 mmol/L, and LVEF <35% or LVEF 35–55% in case of severe mitral regurgitation or aortic stenosis.	No difference in the composite of all-cause mortality, resuscitated circulatory arrest, and implementation of another MCS device at 30 days	50%	31%	13.8%
EURO-SHOCK (2023) [46]	35	VA-ECMO vs. OMT	AMI-CS	Hemodynamic instability and end-organ hypoperfusion, clinical signs of pulmonary congestion, arterial lactate > 2 mmol/L.	No difference in 30-day and 12-month all-cause mortality	43.8%	36%	21%
ECLS-SHOCK (2023) [45]	420	VA-ECMO vs. OMT	AMI-CS	Hemodynamic instability and end-organ hypoperfusion, arterial lactate > 3 mmol/L.	No difference in 30-day all-cause mortality	47.8%	23.4%	11%

Abbreviations: CS, cardiogenic shock; IABP, intra-aortic balloon pump; VA-ECMO, venoarterial extracorporeal oxygenation; OMT, optimized medical therapy; CI, cardiac index; AMI, acute myocardial infarction; NR, not reported.

**Table 4:** Comparative evidence across short-term percutaneous MCS devices in AMI-related cardiogenic shock.

Device	Main RCTs (Year)	Typical SCAI Stage/Severity	Population Highlights	Timing of MCS	Primary Outcome and Trend	Practical Implications
IABP	IABP-SHOCK I (2010) [20]; IABP-SHOCK II (2012; 2019) [22,23]	Mostly B–C (milder shock); lactate often low-to-moderate	AMI-CS undergoing PCI; high pre-randomization arrest (45%) in IABP-SHOCK II; frequent post-PCI placement	Predominantly post-PCI or delayed	No mortality benefit at 30 days, 1 year, or 6 years; neutral secondary outcomes	Not routine in AMI-CS without mechanical complications; consider in mechanical complications or as adjuncts
Impella (CP/5.0)	ISAR-SHOCK (2008) [28]; IMPRESS (2017) [29,30]; DanGer Shock (2024) [32,33]	Predominantly C–D (classic–deteriorating); LV-predominant failure	AMI-CS with PCI; IMPRESS had about 92% pre-randomization arrest; DanGer enrolled STEMI-CS with lactate > 2.5 mmol/L, LVEF < 45%	Early/upfront favored (DanGer); earlier initiation emphasized	ISAR-SHOCK: hemodynamic gain; IMPRESS: no mortality difference; DanGer Shock: lower 180-day mortality vs. standard care	Considered in SCAI C–early D, especially before PCI; greatest benefit before multiorgan failure
VA-ECMO	ECLS-SHOCK I (2019) [42]; ECMO-CS (2023) [44]; EURO SHOCK (2023) [46]; ECLS-SHOCK (2023; 2024 1-yr) [45,47]	Often D–E (deteriorating or extremis); frequent post-arrest	AMI-CS with severe hypoperfusion ± respiratory failure; ECLS-SHOCK had about 78% resuscitated; older cohorts; high crossover	Frequently early vs. conservative; many crossovers to ECMO	No routine survival benefit; hemodynamics improved, but mortality unchanged; signal of harm via complications	Reserved for refractory D–E, severe hypoxemia, or post-arrest; plan LV unloading and limb protection
Combination (ECPELLA; IABP + ECMO)	RCT evidence ongoing (EARLY-UNLOAD neutral; UNLOAD-ECMO ongoing)	D–E with LV distension or closed AV; inadequate pulsatility on ECMO	ECMO recipients with pulmonary congestion, LV distension, and high LVEDP/PCWP	Adjunctive (after ECMO start) or upfront in select centers	Observational data: physiologic benefit; no definitive mortality benefit yet	Used when ECMO-induced afterload causes LV distension/pulmonary edema; careful selection
Other devices (TandemHeart; RV support)	Early small RCTs/series (2006–2008) [54,56]; PROTEK Duo (RV) observational	Selected C–D (LV or RV phenotypes)	TandemHeart: LA–aortic centrifugal support (up to ~5 L/min); PROTEK Duo: RV failure support	Center-dependent; often rescue or bridge	Hemodynamics improved vs. IABP; no mortality advantage; more access complications	Considered in experienced centers for specific phenotypes (LV or isolated RV shock)

Abbreviations: CS, cardiogenic shock; IABP, intra-aortic balloon pump; VA-ECMO, venoarterial extracorporeal oxygenation; OMT, optimized medical therapy; CI, cardiac index; AMI, acute myocardial infarction.

## Data Availability

No new data were created or analyzed in this study.
